# The influence of the rural health security schemes on health utilization and household impoverishment in rural China: data from a household survey of western and central China

**DOI:** 10.1186/1475-9276-9-7

**Published:** 2010-02-23

**Authors:** Wuxiang Shi, Virasakdi Chongsuvivatwong, Alan Geater, Junhua Zhang, Hong Zhang, Daniele Brombal

**Affiliations:** 1 Unit of Epidemiology & Health Statistics, Dali University, Dali 671000, China; 2 Epidemiology Unit, Faculty of Medicine, Prince of Songkla University, Hat Yai, Songkhla 90112, Thailand; 3 Health Human Resources Development Center, Ministry of Health, Beijing 100097, China; 4 Cooperation Development Office of Italian Embassy in China, Beijing 100097, China

## Abstract

**Background:**

The New Rural Cooperative Medical Scheme (NRCMS, voluntary health insurance) and the Medical Financial Assistance (MFA, financial relief program) were established in 2003 for rural China. The aim of this study was to document their coverage, assess their effectiveness on access to in-patient care and protection against financial catastrophe and household impoverishment due to health spending, and identify the factors predicting impoverishment with and without these schemes.

**Methods:**

A cross-sectional household survey was conducted in 2008 in Hebei and Shaanxi provinces and the Inner Mongolia Autonomous Region using a multi-stage sampling technique. Information on personal demographic characteristics, chronic illness status, health care use, household expenditure, and household health spending were collected by interview.

**Results:**

NRCMS covered 90.8% of the studied individuals and among the designated poor, 7.6% had their premiums paid by MFA. Of those referred for hospitalization in the year prior to the interview, 34.3% failed to comply, mostly (80.2%) owing to financial constraints. There was no significant difference in the unmet need for admission between the insured with NRCMS and the uninsured. Before reimbursement, the incidence of catastrophic health payment (household health spending more than 40% of household's capacity to pay) and medical impoverishment (household per capita income falling below the poverty line due to medical expense) was 14.3% and 8.2%, respectively. NRCMS prevented 9.9% of the households from financial catastrophe and 7.7% from impoverishment, whereas MFA kept just one household from impoverishment and had no effect on financial catastrophe. Household per capita expenditure and household chronic disease proportion (proportion of members of a household with chronic illness) were the most important determinants of the unmet need for admission, risk of being impoverished and the chance of not being saved from impoverishment.

**Conclusion:**

The coverage of NRCMS among the rural population was high but not adequate to improve access to in-patient care and protect against financial catastrophe and household impoverishment due to health payment, especially for the poor and the chronically ill. Furthermore, MFA played almost no such role; therefore, the current schemes need to be improved.

## Background

Many low- and middle-income countries rely heavily on patients' out-of-pocket health payments to finance their health care systems[1]. Empirical evidence indicates that the out-of-pocket health payment is the least efficient and most inequitable means of financing health care and prevents people from seeking medical care[2]. If patients require extensive or long-term health care, out-of-pocket health expense may result in financial catastrophe (household health spending more than 40% of household's capacity to pay)[1] or even household impoverishment (household per capita income falling below the poverty line due to medical expense), especially for the poor and vulnerable groups[3,4].

In 2003, the Chinese government initiated the New Rural Cooperative Medical Scheme (NRCMS) to reduce the financial burden on rural residents. It is a government-run, voluntary, community-based, and cost-sharing medical insurance program, covering a county (0.5-1 million people) as a unit. This scheme requires the participating household to pay a part of the premium, which includes a household contribution of 10 Yuan (RMB), a central government subsidy of 20 Yuan and 20 Yuan from the local government per capita per year[5]. Local governments are free to choose the benefit package and administrative arrangement of their own NRCMS according to local needs, thus, benefit packages vary from one county to another[6].

To supplement the NRCMS, the Medical Financial Assistance (MFA) was also established in 2003. The MFA is supposed to provide assistance mainly for the designated poor, who are identified by local governments according to the national extreme and relative poverty lines, to pay the NRCMS premium and cover part of the NRCMS non-reimbursable medical expenses. Additionally, the program is designed to reimburse part of the spending on hospital services that the officially designated catastrophic diseases, such as cancer, may incur on the non-poor. It is financed jointly by the central and local governments[7].

Apart from the reports provided by the offices responsible for NRCMS and other insurance schemes[8], there have been only a few independent and rigorous assessments of the program. The 2006 national survey reported that the number of persons living in poverty (according to definition) had over one year increased by 2.5%, from 13.7% to 16.2% -this increase was primarily due to payment of medical expenses [9], but there were no data on the impact of health insurance. A recent study based on the linkage of registration and claim records indicated that NRCMS reduced medical-spending-related poverty headcount and gap (the mean shortfall of the total population from the poverty line with the non-poor being given a shortfall of zero, expressed as a proportion of the poverty line) by 3.5-3.9% and 11.8-16.4%, respectively. However, the number of successful record linkages was only 1,050 out of 5,380 households registered[4]. Another recent study also documented that the percentage of financial catastrophe among the investigated rural population was 9.0% before and 8.3% after NRCMS was introduced. Nevertheless, that study was confined to 375 households, previously identified to have catastrophe by the claim records [10]. To our knowledge, there are no data from a large-scale household survey that can confirm the above findings. Most of these record-based studies ignored the sick who were unable to get admitted to hospital due to financial constraints, thus giving a biased picture. The studied demographic characteristics did not include the proportion of household members with chronic illness, thus, the effect of existing illness in the household on financial burden was not documented. Therefore, a rigorous evaluation of all rural security schemes, based on a larger-scale household survey, is needed.

In this study, we raised the following questions: (1) what is the extent of current coverage of the two schemes? (2) do the schemes improve access to in-patient care? (3) do the schemes offer protection against financial catastrophe? (4) what socio-demographic variables could predict the level of impoverishment due to health payment with and without the schemes?

Our study focuses on the Western and Central Rural regions of China, which comprise a rural population of approximately 500 million [4], where poverty is more prevalent than in other parts of China [11].

## Methods

### Study design

A cross-sectional, community, household survey was conducted from May to August 2008 in Hebei and Shaanxi provinces, and the Inner Mongolia Autonomous Region, which represent Western and Central China. From each selected province, two NRCMS-implemented counties, with population, area and per capita annual income close to the average provincial values, were chosen. Counties which did not have both insurance schemes available to its residents, or where the schemes had been introduced for less than one and half years, were excluded from selection. In each selected county, all the towns were classified into better-off and worse-off groups, depending on their average annual income, and from each group, one town was randomly selected. In each selected town, all natural villages were grouped into two classes: close, and far, depending on their distance from the town centre. In each class, three natural villages were randomly selected and finally, 45 households from each natural village were chosen by simple random sampling.

### System design of NRCMS and MFA in the sampled areas

The average length of time from the introduction of the schemes to the survey was 3.0 years (range 1.5 to 5.5 years). In five out of six counties, the NRCMS redirected 8 or 9 Yuan out of the 10 Yuan annual per capita premium into the family account for out-patient services. The remaining 1 or 2 Yuan (10 Yuan in the county not covering the family account) were put into a fund for in-patient services, to which both the central and local governments also contributed 40 Yuan per capita per year. The premium in the family account would be used to cover out-patient expenses until it was completely used up by that family. For each hospitalization, the first 50-3000 Yuan, depending on the level of the hospital, had to be paid out of pocket. Above that, the patient would co-pay between 20-80% of the remaining fee, depending on the level of the hospital and the amount of health cost. The maximum amount of reimbursement from the schemes ranged from 20,000 Yuan per household (2 counties) and 15,000 to 20,000 Yuan per capita (4 counties) per year. Reimbursement for hospitalization within the respondent's home county is processed on the day of discharge from hospital. However, for admissions outside the respondent's home county application for hospitalization needs to be approved by the NRCMS management office of county before admission can proceed. Documents related to in-patient services have to be sent to the office for verification within 15 days after discharge. Applicants generally have to wait up to one month for processing and approval before getting reimbursed.

Regarding MFA, all but one county paid the NRCMS premium for the designated poor. In two counties, the MFA did not implement in-patient reimbursement. Residents in the other four counties can have their the NRCMS non-reimbursable in-patient payment reimbursed by MFA, up to a maximum amount of 3,000 to 10,500 Yuan, with a co-payment ranging from 0-50%, depending on the benefit package of their own county. To obtain the MFA reimbursement, the eligible person needs to travel to the Office of County Government to fill out the reimbursement application form. The travel time, in general, takes at least one day. Applicants then wait for at least 3 months for processing and approval before finally traveling back to the office again for reimbursement.

Most of the provider payment methods at the designated clinics and hospitals are fee-for-service. The service fees for a few diseases, such as appendicitis without complication, in two counties, are paid based on the diagnosis-related-group system (DRG).

### Study variables

The study variables and their definitions (where required) are as follows:

#### Personal data

(1) age, (2) gender, (3) ethnicity, (4) education level, (5) occupation, (6) marital status, (7) religion, (8) insurance status, (9) chronic ailment: *An ailment that lasts or is expected to last for at least 12 months, resulting in functional limitations or the need for ongoing medical services, and includes disability*[12], (10) number of episodes of in-patient visits (hospitalizations) within 12 months prior to the survey, (11) unmet in-patient need: *this followed the method of EU-SILC*[13]. A question was asked whether there was any time during the last 12 months when the respondent needed hospitalization as referred by a doctor, but could not get it. If yes, the reason for not being admitted was further sought, with prompted responses including financial barrier, waiting list, distrust in the quality of care provided and so on.

#### Household data

(12) Household per capita expenditure: *although household income information was available, we used total household expenditure, which could be more accurately assessed. Expenditure per capita of each household reflects living standard *[14]. For expenses, a recall period of three months for spending on regular items, and one year for non-regular items was applied and they were categorized as food and non-food expenses.

(13) Health payment: *out-of-pocket health payment as a share of household resources, and including the payment for outpatient care, self-prescribed medication and traditional medicine services with a recall period of one month, and in-patient care payment with a recall period of one year preceding the survey*[15]. Expenses related to non-medical items such as transportation, special foods, and accommodation, were excluded.

(14) Household capacity to pay for services: *household's non-food spending (total household expenditure minus food expenditure)*[1].

(15) Catastrophic health payment: *a condition when the total amount of medical expenditure exceeds 40% of household capacity to pay for services in the past 12 months prior to the interview *[1,16].

(16) Household in poverty: *defined according to the internationally accepted definition of absolute poverty; household per capita income less than 1 USD per day (1,300 Yuan per year per capita in 2008 in China) *[17]. This was higher than the national poverty line of 1,067 Yuan, which is the reason why poverty is underestimated in China [18]. Household poverty occurs when household per capita expenditure (total household expenditure divided by total number of household members) is less than the threshold [14].

(17) Household impoverished by medical expenses: *a household that originally had its per capita expenditure above the poverty line, but which fell below the line after health payment*[14].

### Data Collection

The questionnaire for the household survey was modified from that used in the Fourth National Health Service Survey in 2008 in China to suit the needs of the study[19]. It consisted of six sections: a) household composition, status of the designated poor, status of household participation in NRCMS, household income and expenditure; b) availability of health care facilities to the household; c) age, gender, ethnicity, religion, marital status, highest education level attained, employment status, health insurance status and self-reported chronic illness status of household members; d) health utilization and associated costs occurred within 30 days prior to the interview; e) hospitalization 12 months prior to the interview; and f) the frequency and their causes for not seeking hospitalization within the past 12 months prior to the interview. To validate the questionnaires, input from public and scientific advisory committees was obtained, and 2 pilot studies were conducted before the formal survey.

Thirty six medical students from the College of Medicine at Xianjiaotong University, Hengshui Medical School and Chifeng Medical School were trained to be data collectors under the supervision of three senior experts. In the procedure of data collection, the household head and all other household members aged between 15 and 65 were interviewed. The household head also served as a proxy for household members aged above 65 or below 14. Respondents were given full explanation of the research purpose before being invited to participate and after they gave their informed consent a face-to-face interview was conducted. Data were collected between May and August 2008.

### Data analysis

The characteristics of the studied individuals were summarized in terms of frequency and percentage, or mean and standard deviation. Household per capita expenditure was grouped into quintiles and household chronic disease proportion (defined as the total number of members with chronic illness divided by total number of household members) was divided into 3 levels, i.e. 0, >0 to ≤0.5, >0.5.

The incidence of financial catastrophe due to health payment was calculated by dividing the total number of households facing financial catastrophe by the total number of households under study[14]. The incidence after reimbursement was then calculated by removing the number of households in which catastrophe was avoided from the numerator. The proportion of households in which catastrophe was avoided by the insurance was further expressed as a fraction of all households with catastrophe before reimbursement. The intensity of financial catastrophe due to health payments was defined as the average degree by which health payments(as a proportion of household's capacity to pay) exceed the threshold(40%)[14]. Similar to the analysis of catastrophe incidence, the proportion of the intensity avoided by the insurance was also calculated.

The incidence of medical impoverishment was defined as the total number of households impoverished by health spending divided by the total number of households under study[14]. The intensity of medical impoverishment was defined as the difference in poverty gap between pre-payment and post-payment [14]. The effect of the schemes on preventing these impoverishment parameters was computed in a similar fashion to that of catastrophe analysis.

Logistic regression was applied to identify the determinants of unmet need for hospitalization, household impoverishment before reimbursement and household avoidance of impoverishment after reimbursement. In reference to the analysis of unmet need for in-patient care, the dependent variable was the individuals' unmet admission (the individual who did not need admission as referred by a doctor was excluded) and the independent variables included household per capita expenditure, household chronic disease proportion, insurance status, and demographic characteristics of the individual (age, gender, ethnicity, level of education, occupation). In the two other models, the outcome variables were household impoverishment without reimbursement and household avoidance of impoverishment due to reimbursement, respectively. The two latter models used the household level explanatory variables adopted by the first model, plus the duration of NRCMS and MFA implementation as their predictors.

All data were coded and computerized using EpiData 3.0 software and analyzed with R 2.9 software and Epicalc package [20].

### Ethical considerations

The proposal of this study was approved by the Ethics Committee of the Faculty of Medicine, Prince of Songkla University, Thailand, and further endorsed by the Health and Human Resource Development Center of the Ministry of Health, P.R. of China, before the research was carried out.

## Results

A total of 3,340 households in six counties were selected covering 11,252 individuals, representing a response rate of 99.8%. The mean age of the 9,032 direct respondents was 39.3 years and the male to female ratio was 1.49. The percentage of persons requiring a proxy interview was 19.7%.

Table 1 shows the characteristics of the sampled individuals. Of the 11,252 individuals, 90.8% participated in NRCMS (91.2% of all households) and 8.0% had no insurance (7.8% of all households). Among the designated poor (1,337 individuals), only 7.6% had their premium paid by MFA. In addition, the prevalence of chronic illness among the expenditure groups, in ascending quintile order, was 34.5%, 27.9%, 22.3%, 20.6%, and 19.4%, respectively.

**Table 1:** Characteristics of the sampled individuals (N = 11252)

Variables		n (%)
**Sex**		
Male		5829 (51.8)
Female		5423 (48.2)
**Age**		38.4 (19.7)*
**Ethnicity**		
Han		10761 (95.6)
Mongolian		402 (3.6)
Other		89 (0.5)
**Level of Education**		
None		2608 (23.2)
Primary school		3787 (33.7)
Junior high school		3802 (33.8)
Senior high school and above		1055 (9.4)
**Occupation**		
Unemployed		1299 (11.5)
Farmer		7168 (63.7)
Non-farmer		1313 (11.7)
Student		1472 (13.1)
**Religion**		
None		10208 (90.7)
Buddhism		494 (4.4)
Christianity		240 (2.1)
Other		310 (2.8)
**Marital status**		
Unmarried		3275 (29.1)
Married		7510 (66.7)
Divorced or widowed		467 (4.2)
**Insurance status**		
None		905 (8.0)
NRCMS only		9820 (87.3)
Private insurance only		131 (1.2)
NRCMS + private insurance		396 (3.5)

* Mean and standard deviation

### Hospitalization and unmet in-patient need

A total of 628 patients from 532 households were hospitalized on an average of 1.23 times in the year prior to the interview, yielding an admission rate of 0.07 admissions per capita per annum. For an admission service, the average non-reimbursable fee paid by a household was 1,462.4 Yuan (IQR = 623.9 - 3,249.7 Yuan) or 74.3% of the total hospital spending. The amount of this fee (in Yuan) by expenditure group in ascending quintile order was 491.2, 956.9, 1,076.0, 2,017.8 and 3,377.9, respectively. In reference to non-admission services, which included out-patient care, self-treatment and use of traditional medicine, the non-admission rate was 4.1 times per capita per year, which is significantly higher than that of admission services (p < 0.05), and the average non-reimbursable fee per time paid by a household was 43.8 Yuan (IQR = 16.7 - 102.3 Yuan) or 97.0% of the total non-admission spending.

Of the 11,252 individuals interviewed, 899 (8.0%) were referred for hospital admission during the 12 months prior to the interview. Of these, 308 (34.3%) did not seek in-patient services after the doctor's referral (unmet in-patient need). Of the 820 patients who were enrolled in NRCMS, 285 (34.8%) did not seek in-patient services compared with 29.1% of the 79 patients who were not enrolled (p > 0.05).

The most common reasons for not seeking in-patient services were: economic barriers (80.2%), condition judged not serious (4.5%), and poor service quality or ineffective treatment (3.7%). The proportion of responders who had their need for in-patient admission unmet was inversely related to level of education and increasing household expenditure, and directly associated with proportion of persons in household with chronic illness. With decreasing household income, the unmet need for in-patient care steadily increased from 25.5% in the highest quintile to 54.8% in the lowest quintile (Table 2).

**Table 2:** Summary of unmet need for hospitalization by individual and household characteristics (N = 11252)

Variables	Patients referred for admission n (%) (a, b)	Patients with unmet admission n (%) (c, d)	Adjusted OR and CI 95% (logistic regression)	
			
			OR	95% CI
**Household per capita expenditure (Quintiles)**				
Lowest	115 (5.3) (a1, b1)	63 (54.8) (c1, d1)	4.23	(2.55, 6.96) ^‡^
2nd	126 (5.4) (a2, b2)	42 (33.3) (c2, d2)	1.72	(1.05, 2.81) ^†^
3rd	164 (7.4) (a3, b3)	58 (35.4) (c3, d3)	1.79	(1.15, 2.80) ^†^
4th	220 (10.3) (a4, b4)	75 (34.1) (c4, d4)	1.57	(1.04, 2.36) ^†^
Highest	274 (13.2) (a5, b5)	70 (25.5) (c5, d5)	1.0	Reference
**Household chronic disease proportion**				
0	141 (2.8)	14 (9.9)	1.0	Reference
≤0.5	448 (10.1)	139 (31.0)	4.56	(2.50, 8.32) ^‡^
>0.5	310 (21.5)	155 (50.0)	10.27	(5.55, 18.91) ^‡^
**Education level**				
None or primary school	645 (10.3)	251 (38.9)	1.0	Reference
Junior high school	194 (5.3)	46 (23.7)	0.62	(0.42, 0.92) ^†^
Senior high school or above	60 (6.2)	12 (20.0)	0.49	(0.25, 0.97) ^†^
**Status of participation in NRCMS**				
No	79 (8.5)	23 (29.1)	1.0	Reference
Yes	820 (8.3)	285 (34.8)	1.23	(0.71, 2.12)
**Total**	899 (8.4)	308 (34.3)		

Note: †: significant at 5%; ‡: significant at 1%

Examples of calculation: d1 = (c1/a1) ×100%; d2 = (c2/a2) ×100%; d3 = (c3/a3) ×100%; d4 = (c4/a4) ×100%; d5 = (c5/a5) ×100%.

### Catastrophic health payment

Table 3 compares catastrophic health payment between households with hospitalized members (in-patients) and those who received treatment out of hospital (non-admission) and between those with and without insurance schemes. Non-admission expenditure incurred a larger financial catastrophe (the incidence and intensity were 8.9% and 1.6%, respectively) than admission spending (the incidence and intensity were 2.7% and 0.5%, respectively). For all of the health services combined, the incidence and intensity of catastrophic health payment were 14.3% and 2.8%, respectively. NRCMS reduced those parameters by 9.9% and 16.9%, respectively. However, NRCMS was more effective in decreasing both the incidence and intensity of catastrophic expense for hospital admission (41.6% of the incidence and 49.0% of the intensity) than those for non-hospital care (1.0% of the incidence and 2.5% of the intensity). The differences between the effects of NRCMS alone and the combinations of NRSMS plus the various other insurances were minimal.

**Table 3:** Effects of different types of health service and prepayment scheme on financial catastrophe (N = 3333)

Kind of service	Status of Reimbursement	Incidence of catastrophic health payment		Intensity of catastrophic health payment	
		
		Percentage (a)	Proportion avoided by schemes (%) (b)	Percentage (c)	Proportion avoided by schemes (%) (d)
In-patient and non-admission*	Before reimbursement	14.31 (a0)	Reference	2.84 (c0)	Reference
	After reimbursement				
	-NRCMS	12.90 (a1)	9.9^† ^(b1)	2.36 (c1)	16.9^† ^(d1)
	-NRCMS + MFA	12.87 (a2)	10.1^† ^(b2)	2.35 (c2)	17.3^† ^(d2)
	-all insurances ^Δ^	12.78 (a3)	10.7^† ^(b3)	2.32 (c3)	18.3^† ^(d3)

In-patient Only	Before reimbursement	2.67	Reference	0.51	Reference
	After reimbursement				
	-NRCMS	1.56	41.6^†^	0.26	49.0^†^
	-NRCMS + MFA	1.56	41.6^†^	0.25	51.0^†^
	-all insurances	1.50	43.8^†^	0.24	52.9^†^

Non-admission Only	Before reimbursement	8.88	Reference	1.60	Reference
	After reimbursement				
	-NRCMS	8.79	1.01	1.56	2.50
	-NRCMS + MFA	8.76	1.35	1.56	2.50
	-all insurances	8.76	1.35	1.56	2.50

Note: *: Non-admission payments include the payment for out-patient and self-treatment and traditional medicine service;

Δ: All insurances include NRCMS + MFA + private insurance;

†: Significant at 5%; Examples of calculation:

b1 = (a1-a0)/a0×100%; b2 = (a2-a0)/a0×100%; b3 = (a3-a0)/a0×100%;

d1 = (c1-c0)/c0×100%; d2 = (c2-c0)/c0×100%; d3 = (c3-c0)/c0×100%

### Impoverishment due to health payment

Table 4 provides information on poverty in the studied areas prior to and after the payment for medical costs with and without the various reimbursement sources. Before health payment there were 374 households, or 11.2% of the total, living under the poverty line and the average poverty gap was 3.5%. After payment and prior to or without reimbursement, the percentage of households under the poverty line (poverty headcount) increased to 19.4%, thus 8.2% of all households were impoverished; the poverty gap was widened by 4.0 -7.5%. NRCMS reduced the incidence of medical impoverishment from 8.2 to 7.5%, thus preventing only 7.7% of the households from impoverishment. Similarly, NRCMS lowered the intensity of medical impoverishment by only 10.0% and MFA prevented impoverishment in just one household in this study.

**Table 4:** Effects of health spending and different kinds of insurances on household impoverishment (N = 3333)

	Poverty Indicators					
	
	Household in poverty n (%) (a, b)	Household impoverished by health spending (HIHS) n (%) (c, d)	HIHS prevented by schemes n (%) (e, f)	Poverty gap (%) (g)	Intensity of medical impoverishment (IMI) (%) (h)	Proportion of IMI reduced by schemes (%) (i)
**Original state **(Pre-payment)	374 (11.2) (a0, b0)	Reference (c0, d0)		3.5 (g0)	Reference (h0)	
						
**Post-payment**						
-No reimbursement	646 (19.4) (a1, b1)	272 (8.2)^† ^(c1, d1)	Reference (e0, f0)	7.5 (g1)	4.0^† ^(h1)	Reference (i0)
-Reimbursement by NRCMS	625 (18.8) (a2, b2)	251 (7.6)^† ^(c2, d2)	21 (7.7)^† ^(e1, f1)	7.1 (g2)	3.6^† ^(h2)	10.0^† ^(i1)
-Reimbursement by NRCMS + MFA	624 (18.7) (a3, b3)	250 (7.5)^† ^(c3, d3)	22 (8.1)^† ^(e2, f2)	7.1 (g3)	3.6^† ^(h3)	10.0^† ^(i2)
-Reimbursement by all insurances^Δ^	624 (18.7) (a4, b4)	250 (7.5)^† ^(c4, d4)	22 (8.1)^† ^(e3, f3)	7.1 (g4)	3.6^† ^(h4)	10.0^† ^(i3)

Note: Δ: all insurances included NRCMS+MFA+private insurance

†: significant at 5%;

Examples of calculation:

b0 = (a0/N) ×100%; b1 = (a1/N) ×100%; b2 = (a2/N) ×100%; b3 = (a3/N) ×100%; b4 = (a4/N) ×100%; c1 = a1-a0; c2 = a2-a0; c3 = a3-a0; c4 = a4-a0; d1 = (c1/N) ×100%; d2 = (c2/N) ×100%; d3 = (c3/N) ×100%; d4 = (c4/N) ×100%; e1 = c2-c1; e2 = c3-c1; e3 = c4-c1; f1 = (e1/c1)×100%; f2 = (e2/c1)×100%; f3 = (e3/c1)×100%; h1 = g1-g0; h2 = g2-g0; h3 = g3-g0; h4 = g4-g0; i1 = (h2-h1)/h1×100%; i2 = (h3-h1)/h1×100%; i3 = (h4-h1)/h1×100%.

### Distribution of medical impoverishment and the impact of NRCMS across subgroups

The incidence of medical impoverishment decreased as household expenditure increased (Table 5). Households in the lower expenditure quintiles were more likely to be impoverished by medical payment than those in the higher expenditure quintiles. Among the households impoverished by health spending in the lowest expenditure quintile, NRCMS was able to prevent only 0.7% from poverty, compared to 60% in the richest group. Thus, the rich households were more likely to avoid impoverishment than the poor households; the trend was also true for the intensity of medical impoverishment.

**Table 5:** Distribution of household impoverishment and the impact of NRCMS across all subgroups (N = 3333)

Variables	Poverty Headcount			Poverty Gap		
	
	Pre-payment (%)	* Incidence of medical impoverishment (%)	** Proportion of HIHS avoided by NRCMS (%)	Pre-payment (%)	* Intensity of medical impoverishment (IMI) (%)	** Proportion of IMI reduced by NRCMS (%)
**Household per capita expenditure (Quintiles)**						
Lowest	56.1	21.3	0.7	17.36	14.1	1.9
2^nd^	0.0	12.1	6.2	0.00	3.5	8.6
3^rd^	0.0	3.5	13.8	0.00	1.1	18.2
4^th^	0.0	3.2	38.1	0.00	1.2	42.9
Highest	0.0	0.8	60.0	0.00	0.3	62.0
**Household chronic disease proportion**						
0	12.6	3.8	2.0	3.7	1.7	1.9
≤0.5	9.0	8.3	3.8	2.6	3.8	4.3
>0.5	12.3	19.6	4.3	5.1	10.8	5.2
**Household size (Persons)**						
≤3	11.4	9.5	3.1	4.0	5.3	4.8
4-6	10.9	6.6	3.5	2.8	2.4	5.0
>6	15.4	5.1	0.0	2.7	5.0	0.4
**Duration of NRCMS and MFA implementation**						
<3 years	10.2	7.5	2.8	3.2	3.8	4.3
≥3 years	13.1	9.5	3.8	4.0	4.4	5.7

Note: * Incidence of medical impoverishment and Intensity of medical impoverishment (IMI) equal d and h in Table 4, respectively; ** Proportion of HIHS and IMI reduced by NRCMS equal f and i in Table 4, respectively;

Distribution analysis indicated that all χ^2 ^and ANOVA results were significant (p < 0.05).

### Determinants of medical impoverishment with and without the schemes

From Table 6, statistically significant determinants of impoverishment before reimbursement were household per capita expenditure, household chronic disease proportion, education level of household head, age of household head, employment of household head, and the duration of NRCMS and MFA implementation. Of these variables, the first two had very high odds ratios among the extremely poor households (339.1) and the extremely unhealthy households (16.8). As far as the probability to avoid medical impoverishment after reimbursement is concerned, only the first three determinants were significant. The group with the highest education level had a 45 times higher odds of avoiding falling under the poverty line compared to the uneducated group.

**Table 6:** Determinants of medical impoverishment with and without the schemes (N = 3333)

Variables	Impoverishment before reimbursement		Escaping impoverishment after reimbursement	
	
	OR	95% CI	OR	95% CI
**Household per capita expenditure (Quintiles)**				
Lowest	339.13	(128.52, 894.89)^‡^	0.00	(0, 0.02) ^‡^
2nd	26.9	(10.51, 68.83) ^‡^	0.04	(0, 0.35) ^‡^
3rd	5.07	(1.87, 13.72) ^‡^	0.16	(0.02, 1.76)
4th	4.12	(1.51, 11.24) ^‡^	0.94	(0.09, 9.36)
Highest	1.00	Reference	1.00	Reference
**Household chronic disease proportion**				
0	1.00	Reference	1.00	Reference
≤0.5	4.28	(2.83, 6.47) ^‡^	0.12	(0.02, 0.67) ^†^
>0.5	16.84	(10.30, 27.54) ^‡^	0.04	(0.01, 0.25) ^‡^
**Education level of household head**				
None	1.00	Reference	1.00	Reference
Primary school	0.62	(0.41, 0.94)^†^	3.37	(0.78, 14.55)
Junior high school	0.46	(0.28, 0.74) ^‡^	1.71	(0.26, 11.4)
Senior high school or above	0.38	(0.16, 0.88) ^‡^	45.1	(2.37, 860.2) ^†^
**Age of household head**				
<65	1.00	Reference	1.00	Reference
≥65	2.11	(1.34, 3.32) ^‡^	0.49	(0.11, 2.16)
**Occupation of household head**				
Farmer	1.00	Reference	1.00	Reference
Non-Farmer	1.25	(0.62, 2.52)	1.49	(0.28, 7.96)
Unemployed	1.96	(1.09, 3.52) ^†^	0.48	(0.04, 5.78)
**Duration of NRCMS and MFA implementation**				
<3 years	1	Reference	1.00	Reference
≥3 years	1.54	(1.11, 2.15) ^†^	1.39	(0.45, 4.29)

Note: †: significant at 5% ‡: significant at 1%

## Discussion

In our study, we found that out-of-pocket health expenditure caused financial catastrophe to 14% and impoverishment to 8% of studied households. While NRCMS covered over 90% of all households, it only prevented around 10% of the households from financial catastrophe and 8% from impoverishment. Poorer households and households with chronically ill members had a higher risk of meeting financial catastrophe and being impoverished.

The high coverage rate of NRCMS in this study is consistent with the findings of the Fourth National Health Service Survey in China in 2008 [21] and it is said to be due to its modest premium, the government subsidy, and the strong government mobilization[5].

The proportion of patients with an unmet need for hospitalization in our study is almost the same as that from the study on 27 NRCMS pilot counties in 2005 (34.2%) [8], but higher than that from the national survey in 2008 (20.0%) [21]. The variation in the rate of unmet need for admission may be due to differences in definition of the term and localities among these studies, although they all point to a high prevalence of the problem. An unmet need for admission is more likely present among the poor, the less educated and the chronically ill than other groups, which is similar to the findings of previous studies [22,23], with financial constraint being the main reason (80.2%). Moreover, even in the richest quintile a quarter of the patients had unmet needs, therefore, it is essential to solve the problem of unmet need for admission.

The incidence of catastrophic health payment in the sampled areas of our study was higher than that of a study in a richer province; 14.3% vs. 9.3% [10], but lower than that from the national survey in 2003 (15.8%) [24]. In contrast to our expectations, non-admission expenditure incurred a larger financial catastrophe, in terms of incidence and intensity, than admission expenditure. The hospital admission rate of 0.07 per capita per year in the studied areas was much lower than that of 0.11 per capita per year reported in a study from Thailand[25]. The above phenomenon can be explained by the fact that more than one third of patients forwent admission and sought out-patient, self- or traditional medicine treatment, the cost of which is not reimbursed by NRCMS and other private insurances, leaving the majority of catastrophic payments unassisted.

The incidence of medical impoverishment depends on the definition of the poverty line. Had the Chinese national poverty line been used, the incidence of medical impoverishment would have been 7.5%, compared to 8.2% when using the international poverty line. This is consistent with previous studies, all of which conclude that medical expenditure has become an important source of impoverishment in rural China [4,9,26,27].

NRCMS provides protection against financial catastrophe and household impoverishment due to health spending only to a limited extent, especially among the poorer households and households with chronically ill members- a finding which is consistent with that from other studies [4,10,28]. This is explained by the NRCMS's partial protection against in-patient expenditure and indifference to non-admission spending in the study areas. Thailand, another developing county, is famous for the good performance of its health financing systems in reducing catastrophic and impoverishing health spending, by means of universal health coverage [29]. However, the amount of investment should be taken into account. The Thai government spent 9% of its annual budget on health care in 2008 [30], compared to 2.3% in China in the same year[31]. Recently, the Chinese government has increased its contribution to NRCMS, setting the maximum fund available at 100 Yuan per capita per year in Western and Central regions [32].

The Medical Financial Assistance (MFA) has been conceived as one of the most important mechanisms for improving protection against household impoverishment in the poorest section of the society [7]. Our findings on MFA were different from the promising result of a previous in-house pilot study of the scheme [33]. The current poor performance of the system in our study areas is due to its limited establishment, shallowness of the benefit package, and the complicated procedure for reimbursement which requires a lot of time and money for several round trips, especially for the poor. Therefore, the pitfalls need to be carefully corrected.

There are a number of possible mechanisms underlying the inequity in terms of protection against unmet need for hospitalization and medical impoverishment among income subgroups [34,35]. Firstly, the poor are less likely to receive enough financial help allowing them to be hospitalized when needed, and secondly, since they are close to the poverty line, they are more likely to suffer impoverishment by medical expense and reach a poverty depth that is less likely to be avoided by reimbursement.

Similar to being poor, high household chronic disease proportion is another important risk factor of unmet need for in-patient referral, impoverishment and avoiding impoverishment, each with a dose-response relationship. The causal relationship between self-reported ill-health and impoverishment has been demonstrated in several studies [4,36,37]. Therefore, to reduce financial burden due to health spending, health promotion and prevention needs to be emphasized, financial assistance needs to be specially given to households with a greater proportion of chronically ill members, and the current health schemes should address the specific health needs of the target population with chronic disease.

From our data, education was negatively related to unmet need for admission, and impoverishment, both with and without the schemes. However, the link between education and these variables was less remarkable, compared to the effects of household per capita expenditure and household chronic disease proportion. The most remarkable finding from our study related to education was that households whose head had finished senior high school or above were 45 times more likely to be protected from impoverishment than those whose head was uneducated. All of these findings may suggest that education is a weak predictor for the above-mentioned outcomes and that household heads with a higher level of education may be more successful at finding a way to obtain reimbursement.

Our study has limitations that deserve comment. First, recall on expenditure may be inaccurate, leading to a certain degree of misclassification. Nevertheless, previous studies have suggested that recall on incidence of catastrophic spending and impoverishment barely fades over time[1,9]. Second, the cut-off point for financial catastrophe and household chronic disease proportion was based on an arbitrary value, which may be subject to change [38]. However, the threshold of catastrophe set in this study was similar to that previously accepted, to make it comparable with other studies. Third, indirect health payment data was not available, so the financial consequence of obtaining care may be underestimated. However, we examined direct medical costs because only this part of spending can be reimbursed directly by the NRCMS, which was our main interest. Fourth, the cross-sectional nature of this survey may cause it to suffer from an inability to examine the relationship between the duration of the scheme implementation and its long-term performance in preventing financial hardship due to health expense. Even though the questions were probed one-year retrospectively, this is hardly sufficient for any conclusive findings in that respect. In spite of this inability, we can conclude that high spending on health care is a heavy financial burden to households under our investigation. Finally, we had no data on health care provider behavior related to resource spending, since the system of fee-for-service payment can encourage over-prescribing of drugs, laboratory and imaging tests, and induce over-admission[39].

Nevertheless, the strength of this relatively large-scale fieldwork lies in its capacity to disclose financial problems incurred at the household level in all the steps of the health care system procedures.

## Conclusion

Our study concludes that after an average of three years of implementation, the current schemes barely protected against catastrophic and impoverishing health payment. It is our suggestion that these schemes require greater investment, overall improvement in the benefits packages and inclusion of larger financial assistance to the poor and chronically ill. Our study did not, however, include clinical and hospital-based data. Such data are needed in future studies to give a better understanding on the possible influence the ongoing reforms will have on unnecessary care at different levels of the healthcare service system and how these would influence household financial standing.

## Competing interests

The authors declare that they have no competing interests.

## Authors' contributions

WS was the main person who designed the study, supervised all fieldwork, analyzed and interpreted the data and drafted the manuscript. VC and AG co-supervised WS during study design, analysis and interpretation of data. VC gave further input in manuscript preparation, which was also contributed to by AG. HZ and DB participated in the design and management of fieldwork. JZ conceptualized the project, managed the coordination and oversaw all implementation activities. All authors read and approved the final manuscript.
